# Genomic changes in the biological control agent *Cryptolaemus montrouzieri* associated with introduction

**DOI:** 10.1111/eva.12774

**Published:** 2019-02-11

**Authors:** Hao‐Sen Li, Gerald Heckel, Yu‐Hao Huang, Wei‐Jian Fan, Adam Ślipiński, Hong Pang

**Affiliations:** ^1^ State Key Laboratory of Biocontrol, Ecology and Evolution, School of Life Sciences Sun Yat‐sen University Guangzhou Guangdong China; ^2^ Institute of Ecology and Evolution University of Bern Bern Switzerland; ^3^ College of Life Sciences Tianjin Normal University Tianjin China; ^4^ Australian National Insect Collection, National Research Collections CSIRO Canberra Australian Capital Territory Australia

**Keywords:** biological control introduction, *Cryptolaemus montrouzieri*, evolution, genomics, population genetics

## Abstract

Biological control is the main purpose of intentionally introducing non‐native invertebrate species. The evolutionary changes that occur in the populations of the introduced biological control agents may determine the agent's efficiency and the environmental safety. Here, to explore the pattern and extent of potential genomic changes in the worldwide introduced predatory ladybird beetle* Cryptolaemus montrouzieri*, we used a reduced‐representation sequencing method to analyze the genome‐wide differentiation of the samples from two native and five introduced locations. Our analyses based on a total of 53,032 single nucleotide polymorphism loci showed that beetles from the introduced locations in Asia and Europe exhibited significant reductions in genetic diversity and high differentiation compared with the samples from the native Australian range. Each introduced population belonged to a unique genetic cluster, while the beetles from two native locations were much more similar. These genomic patterns were also detected when the dataset was pruned for genomic outlier loci (52,318 SNPs remaining), suggesting that random genetic drift was the main force shaping the genetic diversity and population structure of this biological control agent. Our results provide a genome‐wide characterization of polymorphisms in a biological control agent and reveal genomic differences that were influenced by the introduction history. These differences might complicate assessments of the efficiency of biological control and the invasion potential of this species but also indicate the feasibility of selective breeding.

## 1 INTRODUCTION

Beneficial organisms, especially invertebrate species, are used in biological control to suppress populations of pests. The intentional introduction of invertebrate species is generally used as a form of biological control (Kumschick, Richardson, & Kueffer, 2013), including classical and augmentative types of control. Classical biological control involves the introduction of non‐native natural enemies into an area for the long‐term control of non‐native pests, whereas the augmentative form is the basis of commercial biological control activities and involves the periodic release of mass‐produced natural enemies that may or may not be native (De Clercq, Mason, & Babendreier, 2011). Similar to other biological introductions or invasions, unexpected evolutionary changes can occur in populations of biological control agents, which might decrease the efficiency of pest control or increase the risk to local environments (Fauvergue, Vercken, Malausa, & Hufbauer, 2012; Guillemaud, Ciosi, Lombaert, & Estoup, 2011). Recently, there have been more reports of undesired side effects on local biodiversity caused by introduced biological control agents through rapid expansion, nontarget attack, and competition, and the invasiveness of these agents is usually related to population genetic changes (e.g., *Harmonia axyridis* in North and South America and Europe, Roy et al., 2016 and *Cactoblastis cactorum*, Zimmermann, Bloem, & Klein, 2004 and *Coccinella septempunctata*, Losey et al., 2012 in North America). Thus, understanding the population genetics of biological control agents can aid in the exploration of their patterns of potential evolutionary changes and may be beneficial for biological control programs.

The mealybug destroyer, *Cryptolaemus montrouzieri* Mulsant (Coleoptera, Coccinellidae), is native to Australia and is a predator specialist of mealybug (Ślipiński, 2007). Over the last century, this predatory ladybird has been introduced to at least 64 countries or regions for classical or augmentative biological control purposes (Kairo, Paraiso, Gautam, & Peterkin, 2013). There is currently only a single available report of the side effects of such introductions on local environments, which showed interference with other biological control agents (Annecke, Karny, & Burger, 1969). However, *C. montrouzieri* populations with different introduction histories can show changes in several life history traits, such as development time, oviposition number, and performance under adverse conditions (Li, Zou, De Clercq, & Pang, 2018). We also detected range expansion in introduced populations in southern China (mentioned by Li et al. 2017). Furthermore, host range tests in a laboratory population revealed nontarget attack abilities (Maes, Grégoire, & De Clercq, 2014). These potential risks, including range expansion and nontarget attack by ladybird populations, might result from evolutionary changes that occurred during and after their introduction.

The evolution of introduced biological control agents is usually explained by processes such as population founding or admixture events, rather than being attributed to phenotypic plasticity or selection (Andraca‐Gómez et al., 2014; Kajita, O'Neill, Zheng, Obrycki, & Weisrock, 2012; Lombaert et al., 2011; Retamal et al., 2016). Furthermore, high genetic differentiation is often observed among introduced populations (Fauvergue et al., 2012). We previously conducted several genetic analyses of native and introduced populations of *C. montrouzieri*; using 12 simple sequence repeat (SSR) markers, we found high and significant genetic differentiation between native and introduced populations (Li et al., 2017). Additionally, we detected signals of positive selection by scanning for nonsynonymous mutations across mitochondrial protein‐coding genes (Li, Liang et al., 2016).

Improvements in next‐generation sequencing (NGS) and bioinformatic tools have spurred the development of genome‐wide genetic markers for studying the ecology and evolution of nonmodel organisms (Davey et al., 2011). Recently, this genomic methodology has been applied for the detection of genetic variation in field and mass‐reared populations of biological control agents and to elucidate the genetic basis of target traits (Lommen, Jong, & Pannebakker, 2017; van de Zande et al., 2014). In this work, we aim to explore the pattern and extent of potential evolutionary changes in worldwide introduced *C. montrouzieri*. We conducted reduced‐representation sequencing of samples from two native and five introduced locations of *C. montrouzieri* and performed a genome‐wide survey of intra‐specific polymorphism. All of the non‐native beetle populations analyzed here result from artificial transfers, and their introduction histories at the studied locations are well documented. Analyses of genomic diversity and structural differences between the populations partially revealed the evolutionary forces functioning during human‐mediated species introduction. Finally, the contributions of these genomic findings to biological control applications are discussed.

## 2 MATERIALS AND METHODS

### 2.1 Sampling and DNA extraction

**Figure 1:**
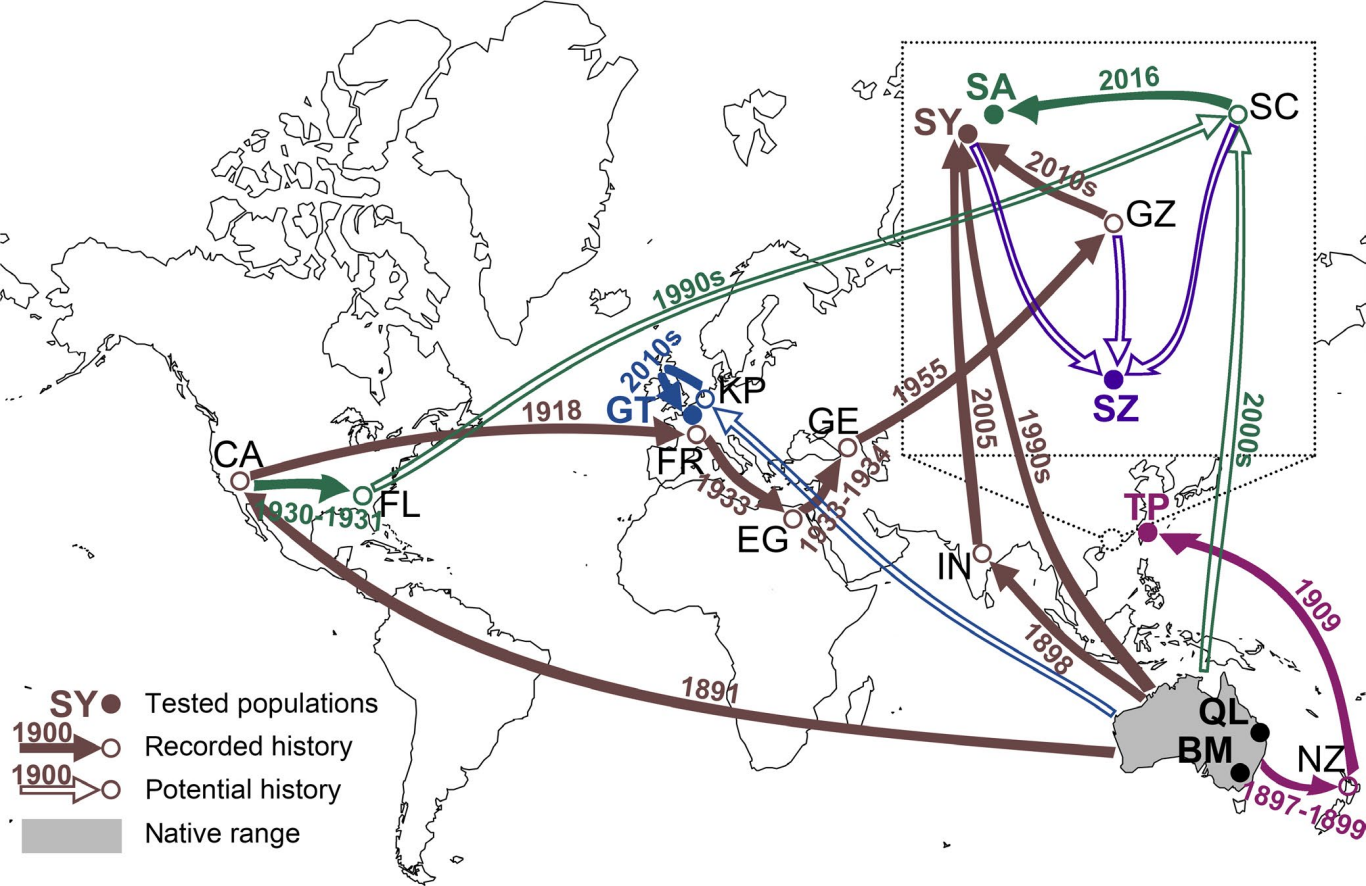
The recorded and potential introduction histories at the studied locations of *Cryptolaemus montrouzieri*. These locations included BM: Canberra, Australia; CA: California; EG: Egypt; FL: Florida; FR: France; GE: Georgia; GT: Ghent University; GZ: Guangzhou; IN: India; KP: Koppert B.V.; NZ: New Zealand; QL: Brisbane, Australia; SC: South China Agricultural University; SY/SA: Sun Yat‐sen University; SZ: Shenzhen; TP: Taipei. Colors represent the histories of introductions including red: SY, green: SA, blue: GT, yellow: SZ, and pink: TP

A total of 128 individuals of *C. montrouzieri* from two native and five introduced locations were analyzed in this study (Figure 1 and Supporting Information Table S1). Individuals from the native locations Brisbane, Queensland, Australia (QL), and Canberra, ACT, Australia (BM), and the introduced locations Shenzhen, mainland China (SZ), and Taipei, Taiwan (TP), were collected in the field. Individuals of SY, SA, and GT were obtained from three cultures in two laboratories at Sun Yat‐sen University (Guangzhou, mainland China) (SY and SA) and Ghent University (Belgium) (GT), respectively. The SY and SA beetles were fed mealybugs, whereas the GT beetles were fed both mealybugs and moth eggs. Laboratory populations are usually mass‐reared in cages under summer conditions (23–27°C and long days) in populations with large numbers (thousands) of individuals, with more than 10 generations generally being produced in 1 year. The introduction histories of the studied locations are shown in Figure 1, which are mainly based on the work of Kairo et al. (2013) and personal communications with the three laboratories. The native sources were recorded for only four studied locations, including Brisbane (QL), Canberra (BM), the Bugs for Bugs biocompany (one of the SY sources), and Queensland (one of the SA sources). GT and TP are assumed to have been founded without population admixture, whereas SY, SA, and SZ appear to have originated from several source populations that were mixed. The dates of introduction ranged from 1909 to 2016. Population sizes were not recorded during or after introduction.

For each individual, total genomic DNA was extracted using a CTAB‐based procedure (Milligan, 1988). DNA quality and quantity were determined using a NanoDrop 1000 spectrophotometer (Thermo Fisher Scientific, USA) and via electrophoretic separation in an agarose gel. Only samples with a DNA concentration >20 ng/μl and slight degradation were used.

### 2.2 Reduced‐representation genome sequencing

Approximately 500 ng of genomic DNA from each sample was processed for reduced‐representation genome sequencing using the specific‐locus amplified fragment sequencing (SLAF‐seq) technique (Sun et al., 2013), with slight modifications. Briefly, in a pilot SLAF experiment, the restriction enzymes Hpy166II and EcoRV‐HF (NEB, USA) were selected because they generate abundant and high‐quality nonrepetitive SLAFs that are relatively evenly distributed across the genome of the model beetle species *Tribolium castaneum* (Richards et al., 2008). In addition, the DNA of a plant species, *Oryza sativa *ssp.* japonica*, was used as a control to assess the normal rate of enzyme digestion. Genomic DNA from each sample was completely digested with these two enzymes, and a single‐nucleotide A overhang was added to the digested fragments. The A‐tailed fragments were then ligated to dual‐index sequencing adapters (Kozich, Westcott, Baxter, Highlander, & Schloss, 2013). PCR was performed in a mixture of diluted restriction–ligation samples including *Taq* DNA polymerase (NEB), dNTPs, and adapter primers. The PCR products were purified using Agencourt AMPure XP (Beckman, UK) and subjected to electrophoresis through a 2% agarose gel. Fragments in the size range of 250–350 bp were excised and purified using a QIAquick Gel Extraction Kit (QIAGEN, Germany). The samples with indexes were allocated to a single lane and subjected to 150 bp paired‐end sequencing on the HiSeq 2500 platform (Illumina, San Diego, USA) at Biomarker Technologies Corporation (China). Real‐time monitoring was performed for each cycle during sequencing. The ratio of high‐quality reads exhibiting Q30 (representing a quality score of 30, indicating a 0.1% chance of an error and, thus, 99.9% confidence) and the guanine‐cytosine (GC) content of the raw reads were calculated for quality control.

### 2.3 SNP calling

Full‐length, high‐quality reads with clear index information were clustered based on their sequence similarity using BLAT v1.9.4 (Kent, 2002), and those sharing >90% identity were grouped to form one SLAF tag. Polymorphic SLAF tags were employed for single nucleotide polymorphism locus (SNP) calling in the GATK v3.7 (McKenna et al., 2010) and SAMtools v1.4.1 (Li et al., 2009) routines. Only SNPs called by both routines were considered to be of high‐quality. The SNPs were further filtered to exclude SNPs present in <80% of individuals and those with a minor allele frequency (MAF) <5% using PLINK v1.90 (Purcell et al., 2007). We excluded potential paralogs by filtering loci with observed heterozygosities (*H*_O_) higher than 0.75 (White, Perkins, Heckel, & Searle, 2013). Only one SNP per SLAF tag was retained to remove the effects of physical linkage. Deviations from Hardy–Weinberg equilibrium (HWE, *p* < 0.05) for each SNP were calculated using PLINK, and those loci that significantly deviated from HWE at more than three studied locations were excluded. The filtered SNPs were mapped to the annotated transcriptome of the species (Li, Pan, De Clercq, Ślipiński, & Pang, 2016; Zhang et al., 2012) using BLASTn, with a cut‐off *E*‐value of 10e‐5 and a minimum length of 70 bp. Finally, the filtered SNPs were converted into Arlequin, BayeScan, FASTA, and PED formats for subsequent analyses using PGDSpider v2.1.0.0 (Lischer & Excoffier, 2012).

### 2.4 Detection of *F*_ST_ outliers

We detected *F*_ST_ outliers among the total SNPs using methods implemented in Arlequin 3.5.1.1 (Excoffier & Lischer, 2010) and BayeScan 2.1 (Foll & Gaggiotti, 2008). We scanned for outlier loci deviating from the expected null distribution of *F*_ST_ in a system of hierarchically structured populations (Arlequin's hierarchical island model, AH, Excoffier, Hofer, & Foll, 2009), which was identified as the best structure in the following analyses (six‐group structure: QL+BM, SY, SA, GT, SZ, and TP). In addition, we used Arlequin's nonhierarchical island model (ANH) to test for *F*_ST_ outliers. In both the AH and ANH analyses, we ran 20,000 simulations, with 10 simulated groups in AH, 100 demes per group, and a maximum expected heterozygosity of 0.5. We also employed the Bayesian *F*_ST_ outlier method implemented in BayeScan to test whether loci were highly differentiated when parameterizing a nonhierarchical island model. BayeScan analysis was performed with a burn‐in period of 50,000, followed by 100,000 Markov chain Monte Carlo (MCMC) iterations. The *q*‐values for each locus in AH and ANH were subsequently calculated from the *p*‐value with *p.adjust* in R to monitor the false discovery rates (FDRs). Those loci with *q*‐values below 0.05 were considered *F*_ST_ outliers. Some simulation studies have suggested that potential error rates must be evaluated by interpreting the results with a variety of methods (de Villemereuil, Frichot, Bazin, Francois, & Gaggiotti, 2014; Narum & Hess, 2011). Thus, we drew Venn diagrams to determine the intersection of high/low *F*_ST_ outliers detected by AH, ANH, and BayeScan. We also drew heatmaps to present the allele frequencies of the high *F*_ST_ outliers. The *F*_ST_ outliers detected by the three methods were used for further analysis.

### 2.5 Genetic diversity and population structure

To detect the contribution of different loci to the diversity of this biological control agent, the total SNPs were further separated into three datasets, which included the high and low *F*_ST_ outliers and the remaining loci (assumed as neutral loci). Genetic diversity and population structure were analyzed based on the total loci, the neutral loci, and the high *F*_ST_ outlier loci.

We employed Arlequin to calculate genetic diversity parameters at each of the studied locations, including the percentage of polymorphic loci (Poly%), nucleotide diversity (Pi), observed (*H*_O_), and expected heterozygosity (*H*_E_). Comparisons of these parameters between native and introduced locations were performed using *t* tests in SPSS 21 (IBM SPSS Statistical, Chicago, USA).

We calculated overall and pairwise *F*_ST_ values using Arlequin, with significance estimated based on 10,000 permutations. Then, population structure was studied through (a) the reconstruction of phylogenetic trees, (b) the estimation of ancestry proportions, and (c) an analysis of molecular variation (AMOVA). Neighbor‐joining (NJ) trees were reconstructed in MEGA6 (Tamura, Stecher, Peterson, Filipski, & Kumar, 2013) using the *p*‐distance between individuals and 1,000 bootstrap replicates. The software ADMIXTURE (Alexander, Novembre, & Lange, 2009) was employed to estimate ancestry proportions using different numbers of ancestral clusters (*K*). We explained the genetic structure by obtaining the lowest cross‐validation error from *K* = 2 to 10. Then, AMOVA was performed in Arlequin with 10,000 permutations to further test for the population structure identified by clustering with ADMIXTURE.

## 3 RESULTS

### 3.1 SNP calling

A total of 504.25 million reads were obtained from the raw sequencing data. The sequencing quality of each sample is shown in Supporting Information Table S2. The number of reads per sample ranged from 1,142,115 to 7,711,651, with an average of 3,920,825 and a standard deviation of 1,396,936. The Q30 of each sample ranged from 93.53% to 96.56%, with an average of 95.38% and a standard deviation of 0.77%. The GC% of each sample ranged from 35.44% to 43.56%, with an average of 36.55% and a standard deviation of 1.13%. The above parameters suggested that all the sequencing data were of high‐quality for further analysis.

Initially, 3,670,447 SNPs were detected among the 1,423,906 SLAF tags, and 53,032 of these loci were retained after filtering for integrity (excluding SNPs present in <80% of individuals), MAF, *H*_O_, a single SNP per tag, and HWE (Table 1). These 53,032 SNPs were further employed for genetic analyses. Among these SNPs, 10,507 were mapped to the annotated transcriptome (Table 1) and were detected in every EuKaryotic Orthologous Groups (KOG) category (Supporting Information Figure S1).

**Table 1:** Procedures and results of SNP calling and filtering

Parameter	Value
SLAF tags	1,423,906
Poly SLAF tags	430,841 (30.26% of total SLAF tags)
Initial SNPs	3,670,447
SNPs present in >80% of individuals	689,925 (18.80% of initial SNPs)
SNPs with MAF >5%	285,397 (7.78% of initial SNPs)
SNPs with *H*_O_ <0.75	285,072 (7.77% of initial SNPs)
One SNP per SLAF tag	53,181 (1.45% of initial SNPs)
SNPs in HWE	53,032 (1.44% of initial SNPs)
Mapped to the annotated transcriptome	10,507

Note

*H*_O_: observed heterozygosity; HWE: Hardy–Weinberg equilibrium; MAF: minor allele frequency; Poly SLAF tags: polymorphic SLAF tags; SLAF: specific‐locus amplified fragment.

### 3.2 Detection of *F*_ST_ outliers

**Figure 2:**
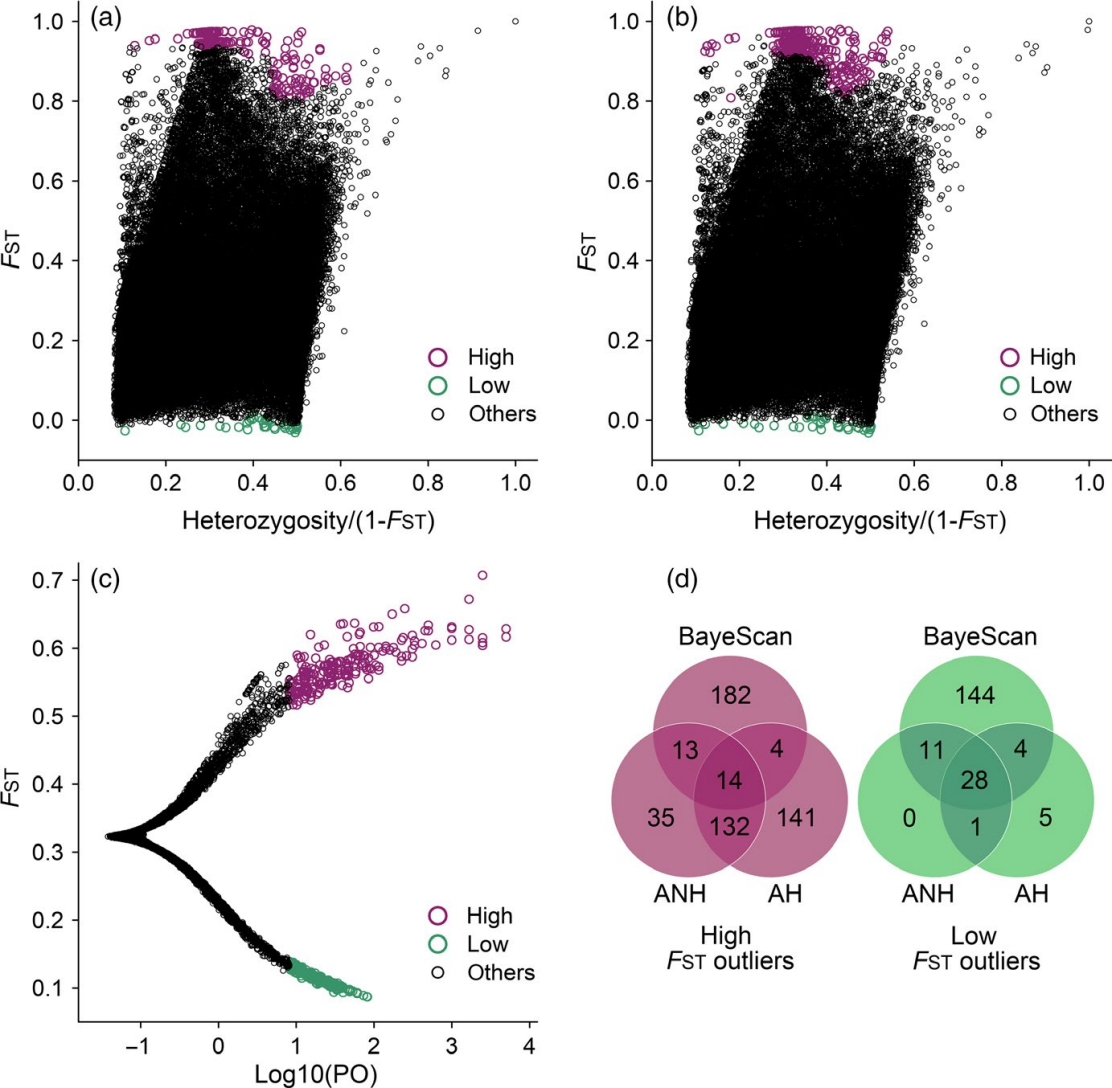
Detection of *F*_ST_ outliers based on three methods. The presented results are locus‐specific *F*_ST_ values against heterozygosity from (a) the hierarchical (AH) and (b) nonhierarchical (ANH) island models implemented in Arlequin, (c) the posterior odds of the selection model (Log10(PO)) and the locus‐specific* F*_ST_ value for each SNP according to BayeScan results. (d) Venn analysis of the above three methods for detecting high and low *F*_ST_ outliers

The three genome scanning methods detected a total of 521 high and 193 low *F*_ST_ outliers (Figure 2). Venn diagrams of the results (Figure 2d) showed that most of the high *F*_ST_ outliers detected by BayeScan were unique, whereas AH and ANH shared many high *F*_ST_ outliers. Among the three methods, 14 high *F*_ST_ outliers were shared. In contrast, 28 of the low *F*_ST_ outliers detected were common to the three methods.

**Figure 3:**
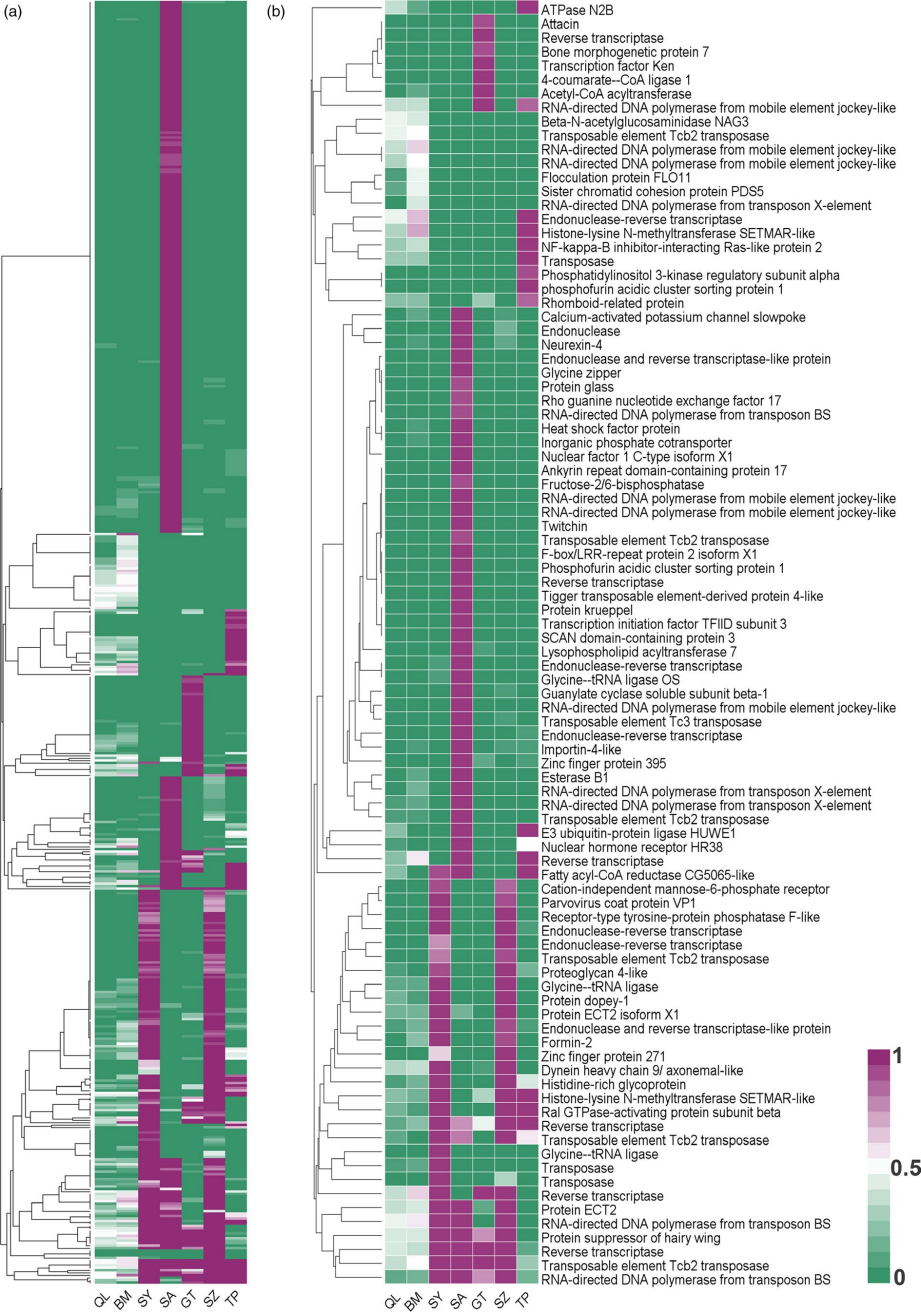
Heatmaps of the normalized allele frequencies of (a) the total 521 high *F*_ST_ outliers and (b) the 92 annotated high *F*_ST_ outliers. Color represents the percentage of minor allele of QL in each population from 0% (blue) to 100% (red)

The allele frequencies of the high *F*_ST_ outlier loci at the seven studied locations showed that most of the high *F*_ST_ outliers were caused by the drastically different allele frequencies in the five introduced populations (Figure 3a). A total of 114 high *F*_ST_ outliers were mapped to genes of the annotated transcriptome (Figure 3b), some of which were related to the food source, including *fructose‐2,6‐bisphosphatas*e (carbohydrate metabolism, with a dramatically different allele frequency in SA), acetyl‐CoA acyltransferase (lipid metabolism, GT), *glycine‐tRNA ligase* (amino acid metabolism, SY/SZ), and the *calcium‐activated potassium channel slowpoke *(inorganic ion transport, SA).

### 3.3 Genetic diversity and population structure

The genetic diversity parameters of each studied location are shown in Table 2 (all 53,032 loci) and Supporting Information Table S3 (52,318 neutral loci and 521 high *F*_ST_ outlier loci). The results based on the datasets containing all loci and the neutral loci only were similar, while the results based on the high *F*_ST_ outlier loci were quite different. Significant reductions in the number of polymorphic loci and nucleotide diversity were found when the introduced populations were compared to the native populations (*p* values ranged from 0.002 to 0.003 in Poly% and from 0.007 to 0.026 in Pi in the three datasets according to *t* tests). There was no significant difference in *H*_O_ and *H*_E_ values between the native and introduced locations.

**Table 2:** Genetic diversity at seven studied locations based on all 53,032 loci, including the percentage of polymorphic loci (Poly%), nucleotide diversity (Pi), observed heterozygosity (*H*_O_), expected heterozygosity (*H*_E_)

Location	Poly%	Pi	*H*_O_	*H*_E_
QL	89.1	0.244	0.229	0.274
BM	89.3	0.284	0.368	0.319
SY	61.4	0.195	0.372	0.318
SA	45.3	0.125	0.322	0.276
GT	63.9	0.205	0.297	0.320
SZ	59.8	0.189	0.309	0.315
TP	51.0	0.194	0.382	0.380
*p* in *t*‐test	0.002*	0.026*	0.452	0.442

Note

BM: Canberra, ACT, Australia; GT: Ghent University (Belgium); QL: Brisbane, Queensland, Australia; SY/SA: Sun Yat‐sen University; SZ: Shenzhen; TP: Taipei, Taiwan.

* Significant differences in means between native QL/BM and introduced SY/SA/GT/SZ/TP according to *t* tests.

**Figure 4:**
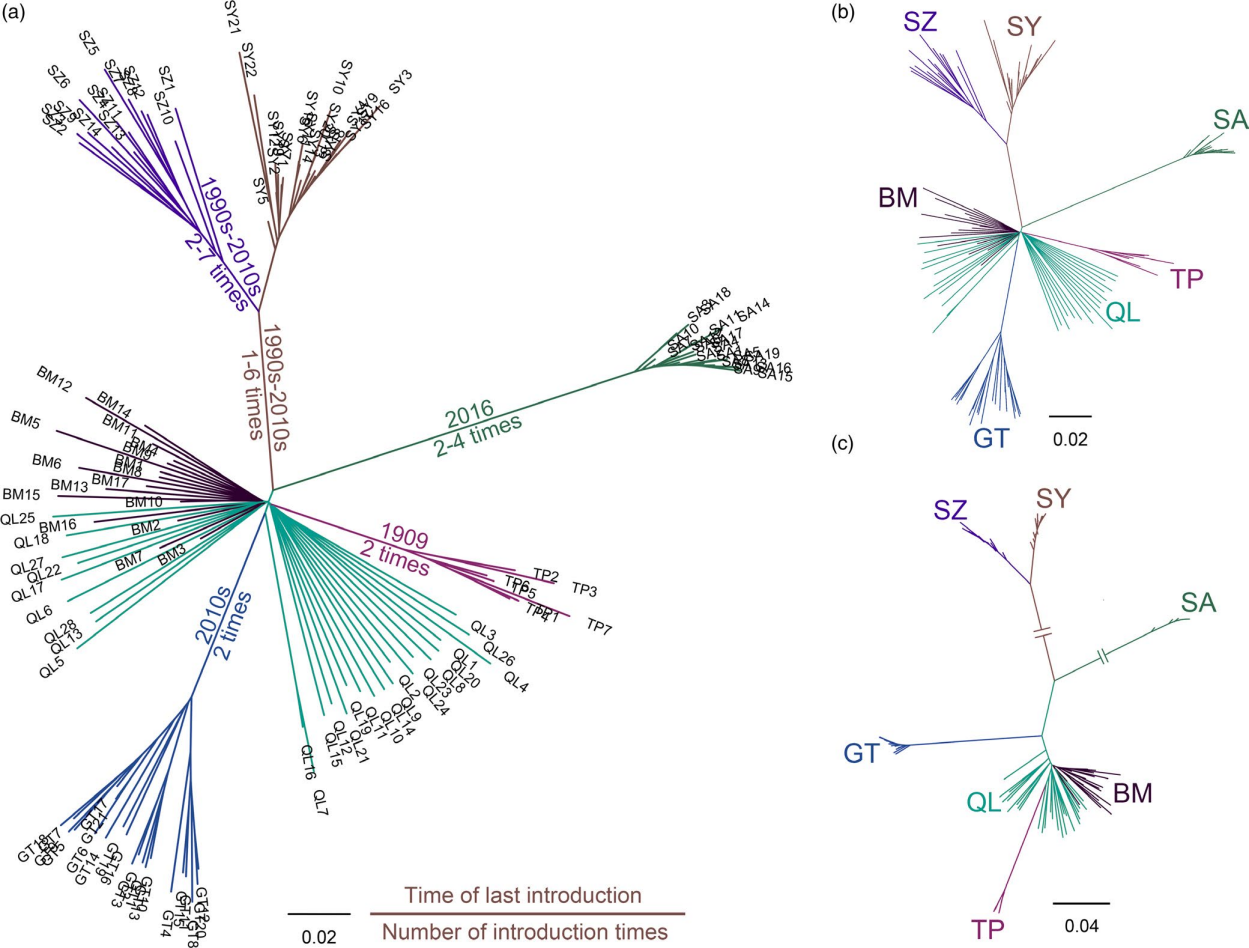
Neighbor‐joining trees of *Cryptolaemus montrouzieri* individuals based on (a) all 53,032 loci, (b) 52,319 neutral loci, and (c) 521 high *F*_ST_ outlier loci. Information including the time of the last introduction and the number of introduction events for each introduced population is provided on the tree branches

The inferred population structure among *C. montrouzieri* samples was very similar between the dataset with all loci and only the neutral loci. In the dataset with all loci, we detected high differentiation among global populations (overall *F*_ST_ = 0.323). The pairwise *F*_ST_ values among the studied locations are shown in Table 3. The two native samples from Australia exhibited a low level of differentiation (*F*_ST_ = 0.017, *p* < 0.001), while all introduced populations showed much higher differentiation compared to the native populations (*F*_ST_ values from 0.113 to 0.319, *p* < 0.001). All of the introduced populations were also highly differentiated from each other (*F*_ST_ values from 0.207 to 0.495, *p* < 0.001). The NJ tree clustered individuals largely by population of origin, placing individuals from the native Australian range (QL, BM) in the middle of separate branches leading to the introduced populations SA, GT, and TP as well as the common branch to SY and SZ (Figure 4a).

**Table 3:** Pairwise *F*_ST_ based on all 53,032 loci. All* F*_ST_ values were significantly different for zero with *p* < 0.001

	QL	BM	SY	SA	GT	SZ	TP
QL							
BM	0.017						
SY	0.213	0.188					
SA	0.319	0.315	0.465				
GT	0.181	0.172	0.348	0.453			
SZ	0.225	0.194	0.207	0.495	0.361		
TP	0.126	0.113	0.309	0.456	0.288	0.327	

Note

BM: Canberra, ACT, Australia; GT: Ghent University (Belgium); QL: Brisbane, Queensland, Australia; SY/SA: Sun Yat‐sen University; SZ: Shenzhen; TP: Taipei, Taiwan.

**Figure 5:**
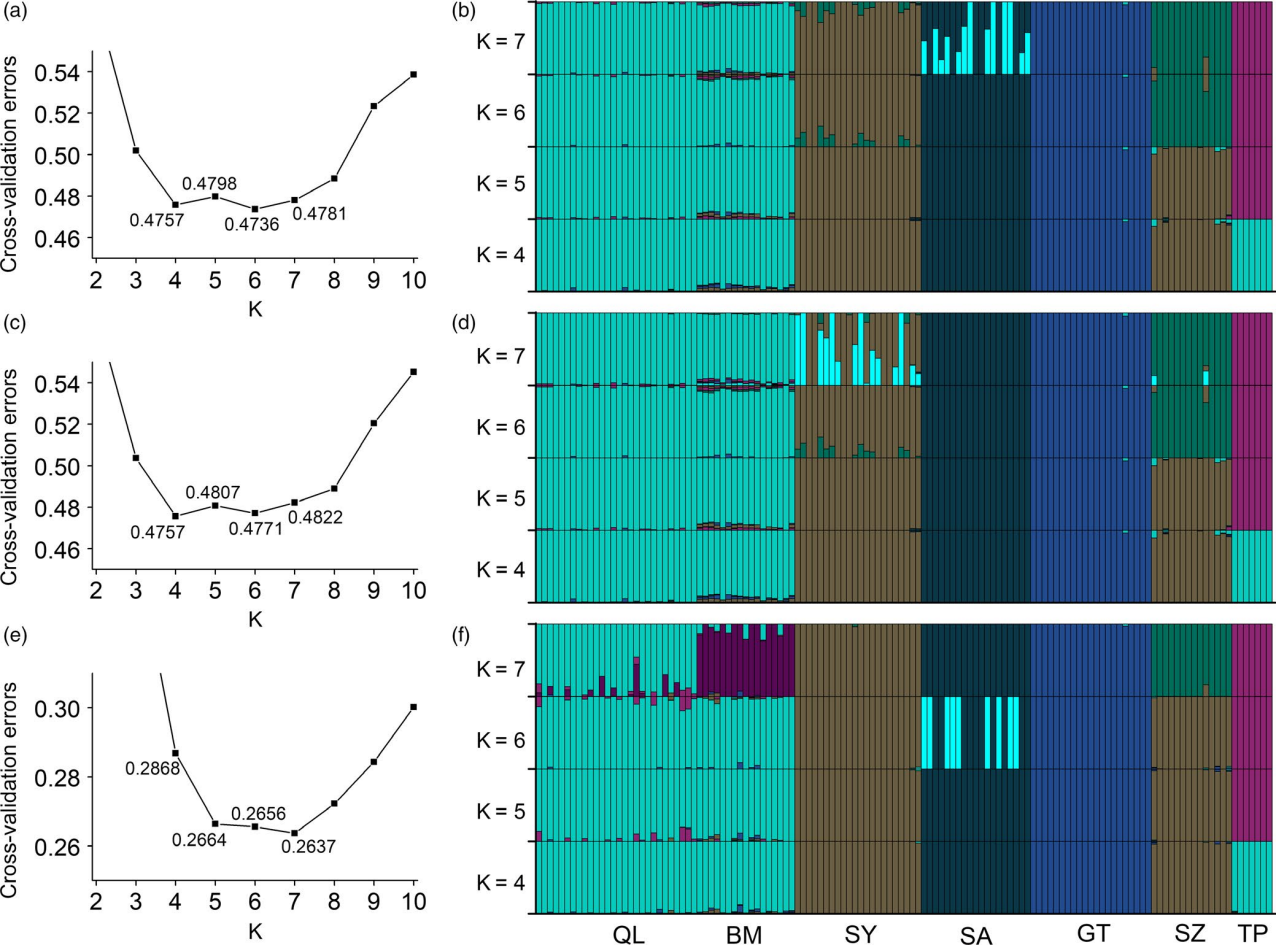
Ancestral clustering using ADMIXTURE based on all 53,032 loci (a, b), 52,318 neutral loci (c, d), and 521 high *F*_ST_ outlier loci (e, f). The cross‐validation error of different numbers of ancestral clusters (*K*) and the individual ancestry bar plots for *K* = 4–7 are shown

ADMIXTURE analysis suggested that most of the tested individuals mainly comprised one ancestry. We obtained the lowest cross‐validation error value for *K* = 6 (Figure 5a), with individuals from each introduced population belonging to a unique ancestral cluster, while those from the two native locations shared one genetic cluster (Figure 5b). However, the cross‐validation error values for *K* = 4–7 were quite close. For *K* = 5, SY and SZ shared the same ancestral cluster, while for *K* = 4, QL+BM and TP were also grouped together. For *K* = 7, SA appeared to be an admixed population of two ancestral clusters. However, a too‐large *K* value may have led to an overfitting effect, which forced the algorithm to subdivide the populations. The percentage of variation associated with differences among groups (*F*_CT_) was higher and significant for *K* = 6 structuring (26.94%, *p* = 0.0489, Supporting Information Table S4) than for *K* = 5 (1.92%, *p* = 0.4360, Supporting Information Table S4) and *K* = 4 (4.29%, *p* = 0.2776, Supporting Information Table S4). However, these AMOVAs had limited power since some of the groups were formed by one location only.

The high *F*_ST_ outlier loci generated a population structure that was in many aspects qualitatively similar but quantitatively stronger. The NJ tree showed that SA and SY+SZ were extremely divergent from the other populations (Figure 4c), with *F*_ST_ values ranging from 0.884 to 0.966 between SA and the other populations and from 0.613 to 0.960 between SY+SZ and the other populations (Supporting Information Table S5). The ADMIXTURE analysis showed that *K* = 7 resulted in the lowest cross‐validation error (Figure 5e), with the individuals from the same locations belonging to a unique cluster. For *K* = 6, the subdivision of SA was possibly an artefactual effect. The AMOVAs showed that the *K* = 5 and *K* = 4 structuring explained nearly none of the total variance among populations (Supporting Information Table S4).

## 4 DISCUSSION

### 4.1 Genomic changes in biological control agents arising from introduction

In this study, we demonstrated very high genetic differentiation among introduced populations of the ladybird beetle *C. montrouzieri*. Our results based on a large number of genome‐wide SNP markers are overall consistent with the differentiation patterns in this system detected with a few SSR markers (Li et al., 2017). More generally, high genetic differentiation based on traditional genetic markers (e.g., SSRs) is commonly detected among different introduced populations of biological control agents (Lombaert et al., 2014; Retamal et al., 2016; Sethuraman, Janzen, & Obrycki, 2015). In theory, genetic differentiation among populations can be largely attributed to genetic drift or selection, and the repeated process of establishment of introduced populations from native ones or other introduced populations may contribute to the strength of these effects (Fauvergue et al., 2012). Because environmental conditions (e.g., temperature and diet) differ from native conditions of *C. montrouzieri*, we considered that not only genetic drift but also selection might have contributed to the observed differences between populations. Our reduced‐representation genomic sequencing approach provides a relatively powerful dataset for a better understanding of the extent and the forces contributing to the genetic changes in the introduction history of this biological control agent.

### 4.2 Role of genetic drift and selection in genomic population changes

The studied introduced populations were artificially transferred from the native areas in Australia or introduced populations on other continents. This intentional introduction process shows some similarities to biological invasions (Blackburn, Lockwood, & Cassey, 2015) but could potentially exhibit even more extreme founder effects due to the total isolation of the introduced populations from the native populations. In this study, a significant decrease in genetic diversity was detected in the introduced populations, which is consistent with founder effects together with strong genetic drift during the early stages of introduction. Additionally, our pairwise *F*_ST_ results, NJ trees, and clustering results supported the notion that all the introduced populations are deeply divergent from the native populations. Since these patterns are very similar in the datasets of all loci and only neutral loci, we suggest that genetic drift—in addition to allele frequency changes during the founding event—was the main force shaping the genetic diversity and population structure of the introduced populations of this biological control agent. Only ~100 years has passed since the introduction of *C. montrouzieri* for biological control was initiated (Kairo et al., 2013), but the production of more than 10 generations per year can contribute to rapid genomic changes. We also found that some alleles in the introduced populations were not present in the native locations. Apart from probably rare de novo mutations, it is possible that these alleles are too rare to have been picked up in the limited sampled individuals within a population. An alternative explanation is that some of these genomic differences go back to naturally existing differentiation in the native range of this species, rather than being caused by human activities. Our analyses of native samples from two distinct locations approximately 1,240 km apart showed only very slight genomic differentiation—particularly in relation to the introduced populations. It would require a detailed survey of the natural range (Zemanova, Broennimann, Guisan, Knop, & Heckel, 2018; Zemanova, Knop, & Heckel, 2016) to determine the full extent of natural genetic variation and differentiation but given all other factors, most of the genetic differences detected here arose most likely during and/or after introduction.

Individuals introduced into new areas are usually subject to different environmental conditions and selection pressures than in their native ranges. Thus, signals of selection in fitness‐related traits/genes should be expressed during the local adaptation process. Recently, *F*_ST_ outlier methods have been widely used in population genomic analyses for detecting candidate loci under selection (Carreras et al., 2017; Jeffery et al., 2017; Pais, Whetten, & Xiang, 2017; Van Wyngaarden et al., 2017). In this study, we detected several high and low *F*_ST_ outliers through three alternative methods. High *F*_ST_ outliers are usually considered candidate loci or linked to loci under positive selection, while low *F*_ST_ outliers might indicate negative or balancing selection (Beaumont & Nichols, 1996; Fischer, Foll, Heckel, & Excoffier, 2014). However, with the increase in differentiation caused by genetic drift, loci with higher *F*_ST_ values can be mistaken for evidence of positive selection, since the two mechanisms produce similar signals in genomic variation (Cruickshank & Hahn, 2014; Currat et al., 2006; Vera, Díez‐del‐Molino, & García‐Marín, 2016). In our case, the detected high *F*_ST_ outliers should be considered as only very tentative evidence for positive selection because of the confounding effects of very high differentiation among the studied locations. Nevertheless, some of the high *F*_ST_ outliers were mapped to genes related to functions such as nutrient transport and metabolism. Irrespective of whether the extreme changes in allele frequencies at these loci were caused by genetic drift or positive selection or both, the number and extent of genetic differences between populations make it unlikely that phenotypic traits of these beetles remained completely unaffected.

### 4.3 Implications for biological control

The extent of heritable phenotypic differences between the introduced or native populations of *C. montrouzieri* has not been characterized at a larger scale, but there is likely variation in the performance as a biological control agent. In fact, ladybirds from the SY population have a significantly longer developmental time and perform better under cold stress but worse under starvation than beetles from SA when tested under controlled laboratory conditions (Li et al., 2018). The large genetic differentiation between these and among the other populations indicates several potential issues related to biological control applications.

First, intra‐specific variation might complicate the assessment of biological control efficiency and invasion potential. The evolution of biological control agents not only influences their effectiveness in pest control but also generates potential side effects on local biodiversity (Roderick, Hufbauer, & Navajas, 2012). Such changes could certainly result from phenotypic plasticity that enables ladybirds to respond to varying environments (Murren et al., 2015). However, relatively often, the successful establishment of introduced species has been associated with rapid genetic changes. These genetic changes could be caused by genetic drift, as most likely in the present study, and/or adaptive responses to novel selection pressures (Fauvergue et al., 2012). For example, in a parasitoid wasp introduced to New Zealand from South America for the control of weevil pests, changes in biotype frequency and better performance were detected after 10 years, indicating local adaptation (Phillips et al., 2008). Further studies should take the potential for intra‐specific variation and different reactions within *C. montrouzieri* into account.

Second, the extent of the detected genetic changes might indicate the feasibility of selective breeding for improvement of traits of interest (Lommen et al., 2017). Using methods such as genome‐wide association studies (GWAS), it is possible to identify and characterize loci affecting target traits for further genomic‐based selection (Visscher, Brown, Mccarthy, & Yang, 2012). For example, similar population genomic techniques are being applied to parasitoid wasps, predatory bugs, and predatory mites in the Breeding Invertebrates for Next Generation BioControl Training Network (BINGO‐ITN, http://www.bingo-itn.eu/en/bingo.htm). Our genome‐wide description of polymorphisms in the ladybird *C. montrouzieri* demonstrates ample genetic variation and thus suggests that such approaches might be promising avenues for further improvements of the performance of this biological control agent.

## CONFLICT OF INTEREST

None declared.

## DATA ARCHIVING STATEMENT

All raw sequences are available in the NCBI SRA databases (BioProject: PRJNA445430, BioSample: SAMN08784023–SAMN08784122 and SAMN09702120–SAMN09702148). The filtered 53,032 SNPs used in this study are available at Dryad (https://doi.org/10.5061/dryad.qg215qn).
