# Supplementary figures and images for: Genomic changes in the biological control agent Cryptolaemus montrouzieri associated with introduction
Source: Evol Appl. 2019 Feb 11;12(5):989–1000. doi: 10.1111/eva.12774 (PMC6503826; doi:10.1111/eva.12774)

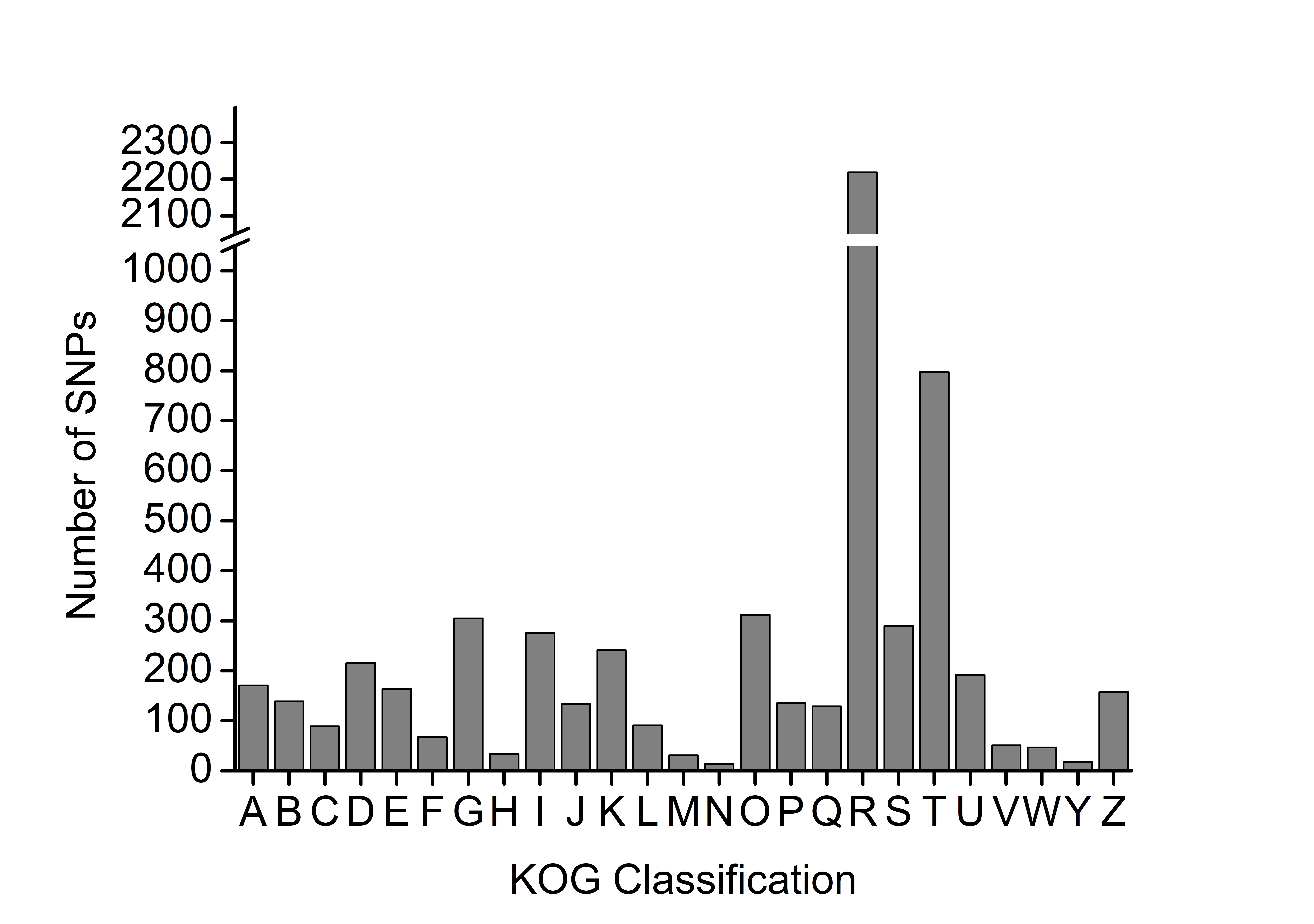

Supplement: Supplementary file 1 [file EVA-12-989-s001.tif]
