# Supplementary material for: Genomic changes in the biological control agent Cryptolaemus montrouzieri associated with introduction
Source: Evol Appl. 2019 Feb 11;12(5):989–1000. doi: 10.1111/eva.12774 (PMC6503826; doi:10.1111/eva.12774)
Supplement: Supplementary file 2 [file EVA-12-989-s002.docx]

**Supplementary tables**

Table S1 Information on the studied locations, including the number of individuals, their sampling locations and types. Specifically, SY and SA were reared in the same laboratory, but they have different histories of introduction and have not been mixed.

| Code | No. of  individuals | Location | Type |
| --- | --- | --- | --- |
| QL | 28 | Brisbane, Australia | Native |
| BM | 17 | Canberra, Australia | Native |
| SY | 22 | Guangzhou, mainland China | Introduced, laboratory-reared |
| SA | 19 | Guangzhou, mainland China | Introduced, laboratory-reared |
| GT | 21 | Ghent, Belgium | Introduced, laboratory-reared |
| SZ | 14 | Shenzhen, mainland China | Introduced, wild-established |
| TP | 7 | Taipei, Taiwan | Introduced, wild-established |

Table S2 Sequencing quality for each studied individual. Aver-depth: average sequencing depth of each SLAF-taq. Heter %: percentage of heterozygous SNP loci.

| Individual | Total reads | GC% | Q30% | Aver-depth | Integrity % | Heter % |
| --- | --- | --- | --- | --- | --- | --- |
| QL1 | 5,882,520 | 36.38 | 93.89 | 19.31 | 45.45 | 7.46 |
| QL2 | 6,305,655 | 35.72 | 94.37 | 21.27 | 44.30 | 7.62 |
| QL3 | 6,617,216 | 35.84 | 94.24 | 23.23 | 42.69 | 7.40 |
| QL4 | 3,052,030 | 35.44 | 94.22 | 13.64 | 31.85 | 5.54 |
| QL5 | 4,841,866 | 35.80 | 93.78 | 19.93 | 35.32 | 6.31 |
| QL6 | 7,311,701 | 35.62 | 94.36 | 26.14 | 41.89 | 6.78 |
| QL7 | 5,507,220 | 35.76 | 94.01 | 22.37 | 36.71 | 5.14 |
| QL8 | 6,933,109 | 35.45 | 93.98 | 24.11 | 43.17 | 7.08 |
| QL9 | 6,613,657 | 35.59 | 93.78 | 23.02 | 42.56 | 7.25 |
| QL10 | 6,773,328 | 35.55 | 94.09 | 23.94 | 42.52 | 7.09 |
| QL11 | 8,146,697 | 35.79 | 94.37 | 28.06 | 43.78 | 7.19 |
| QL12 | 7,711,651 | 35.67 | 94.40 | 26.99 | 43.02 | 7.30 |
| QL13 | 5,877,210 | 36.12 | 93.83 | 20.40 | 43.66 | 7.43 |
| QL14 | 5,910,618 | 35.90 | 93.53 | 20.41 | 43.24 | 7.48 |
| QL15 | 4,475,837 | 35.81 | 93.55 | 16.19 | 41.65 | 7.21 |
| QL16 | 6,901,579 | 36.05 | 94.40 | 24.45 | 43.10 | 6.89 |
| QL17 | 4,378,409 | 35.77 | 94.33 | 16.26 | 40.89 | 7.08 |
| QL18 | 6,860,762 | 36.01 | 93.91 | 24.04 | 44.36 | 7.59 |
| QL19 | 6,008,649 | 36.73 | 93.67 | 20.06 | 45.89 | 8.06 |
| QL20 | 6,487,349 | 36.63 | 93.54 | 21.57 | 46.20 | 7.68 |
| QL21 | 4,881,139 | 35.86 | 93.90 | 18.03 | 41.20 | 7.11 |
| QL22 | 5,036,969 | 36.13 | 94.04 | 17.67 | 43.41 | 8.08 |
| QL23 | 5,665,912 | 36.24 | 93.81 | 19.68 | 44.24 | 7.35 |
| QL24 | 5,497,699 | 36.06 | 94.36 | 19.33 | 43.51 | 7.08 |
| QL25 | 5,417,782 | 36.16 | 94.59 | 19.29 | 43.67 | 7.42 |
| QL26 | 4,770,461 | 35.90 | 94.58 | 16.76 | 43.22 | 7.08 |
| QL27 | 4,712,743 | 36.09 | 94.47 | 16.95 | 42.86 | 7.27 |
| QL28 | 5,908,571 | 35.73 | 94.83 | 21.25 | 43.33 | 7.42 |
| BM1 | 2,655,615 | 36.18 | 95.75 | 8.65 | 45.70 | 13.16 |
| BM2 | 3,227,437 | 35.92 | 95.53 | 10.31 | 46.87 | 14.13 |
| BM3 | 3,637,003 | 36.32 | 95.72 | 11.21 | 48.2 | 15.02 |
| BM4 | 3,171,645 | 36.33 | 95.82 | 10.23 | 46.55 | 14.04 |
| BM5 | 4,013,530 | 36.28 | 95.44 | 14.95 | 42.83 | 7.33 |
| BM6 | 5,599,607 | 36.20 | 95.58 | 19.19 | 46.88 | 8.45 |
| BM7 | 3,256,665 | 36.32 | 94.75 | 10.71 | 45.62 | 13.24 |
| BM8 | 3,047,016 | 36.32 | 95.37 | 9.99 | 46.11 | 13.31 |
| BM9 | 3,298,524 | 36.22 | 95.46 | 10.64 | 46.84 | 13.55 |
| BM10 | 3,405,007 | 36.25 | 94.85 | 10.42 | 48.27 | 14.72 |
| BM11 | 2,433,354 | 36.44 | 95.51 | 8.38 | 43.91 | 13.12 |
| BM12 | 1,142,115 | 36.28 | 95.34 | 4.84 | 35.53 | 8.72 |
| BM13 | 1,527,739 | 36.33 | 95.02 | 5.64 | 39.51 | 11.08 |
| BM14 | 1,734,772 | 36.40 | 95.60 | 6.01 | 41.91 | 12.22 |
| BM15 | 1,586,423 | 36.80 | 95.30 | 4.81 | 35.85 | 8.72 |
| BM16 | 2,141,830 | 36.35 | 95.81 | 7.53 | 41.68 | 9.91 |
| BM17 | 2,877,856 | 36.24 | 95.54 | 9.62 | 45.25 | 13.37 |
| SY1 | 5,105,532 | 36.59 | 95.88 | 17.07 | 47.21 | 7.54 |
| SY2 | 4,213,227 | 37.08 | 95.51 | 14.43 | 45.67 | 8.35 |
| SY3 | 3,556,111 | 37.03 | 95.83 | 14.00 | 41.14 | 3.87 |
| SY4 | 4,089,914 | 36.75 | 95.83 | 14.83 | 43.09 | 5.72 |
| SY5 | 3,957,465 | 36.54 | 95.81 | 13.08 | 46.93 | 9.15 |
| SY6 | 4,241,139 | 36.39 | 95.88 | 14.78 | 45.63 | 6.87 |
| SY7 | 3,420,700 | 36.31 | 96.11 | 11.71 | 45.06 | 7.78 |
| SY8 | 4,345,327 | 36.46 | 96.26 | 13.76 | 46.82 | 7.07 |
| SY9 | 2,914,705 | 36.17 | 95.92 | 10.42 | 40.95 | 5.33 |
| SY10 | 3,405,530 | 36.26 | 95.97 | 12.79 | 42.56 | 5.13 |
| SY11 | 3,288,645 | 36.38 | 95.79 | 11.72 | 44.06 | 7.35 |
| SY12 | 3,379,562 | 36.26 | 95.73 | 11.95 | 43.25 | 7.04 |
| SY13 | 3,837,235 | 36.41 | 95.41 | 13.03 | 46.39 | 7.19 |
| SY14 | 2,956,839 | 36.29 | 95.97 | 10.88 | 42.54 | 7.70 |
| SY15 | 4,241,902 | 36.25 | 96.01 | 14.83 | 45.23 | 6.31 |
| SY16 | 3,773,864 | 36.65 | 95.96 | 12.62 | 42.69 | 5.26 |
| SY17 | 3,670,777 | 36.35 | 95.92 | 13.43 | 43.54 | 6.13 |
| SY18 | 4,144,274 | 36.56 | 95.86 | 13.35 | 46.87 | 7.28 |
| SY19 | 4,002,199 | 36.74 | 95.74 | 13.73 | 45.38 | 7.60 |
| SY20 | 4,485,947 | 36.31 | 96.25 | 15.53 | 45.72 | 7.01 |
| SZ21 | 4,455,103 | 38.33 | 95.20 | 23.52 | 22.99 | 4.02 |
| SZ22 | 2,914,270 | 37.44 | 95.85 | 12.19 | 35.39 | 4.42 |
| SA1 | 2,665,435 | 36.59 | 95.61 | 9.56 | 39.54 | 4.38 |
| SA2 | 3,236,519 | 36.18 | 96.02 | 11.79 | 41.55 | 5.00 |
| SA3 | 3,697,884 | 36.35 | 95.83 | 12.52 | 42.21 | 4.45 |
| SA4 | 1,738,004 | 36.17 | 95.87 | 7.54 | 35.66 | 4.38 |
| SA5 | 2,601,430 | 35.82 | 96.08 | 10.15 | 38.29 | 3.89 |
| SA6 | 3,347,215 | 35.97 | 96.10 | 12.59 | 41.17 | 4.80 |
| SA7 | 3,798,468 | 36.10 | 95.94 | 13.64 | 42.33 | 5.53 |
| SA8 | 2,846,780 | 36.37 | 95.86 | 10.58 | 39.54 | 4.03 |
| SA9 | 4,285,747 | 36.30 | 96.05 | 14.38 | 41.65 | 3.92 |
| SA10 | 3,706,193 | 36.14 | 96.18 | 12.63 | 42.44 | 5.60 |
| SA11 | 3,780,661 | 36.68 | 95.98 | 11.57 | 41.38 | 4.33 |
| SA12 | 3,533,017 | 36.55 | 95.92 | 12.49 | 42.12 | 5.01 |
| SA13 | 2,596,365 | 39.90 | 95.08 | 8.97 | 38.04 | 3.61 |
| SA14 | 3,440,932 | 43.56 | 95.11 | 13.70 | 35.34 | 3.03 |
| SA15 | 3,538,524 | 36.58 | 96.02 | 13.87 | 39.46 | 2.52 |
| SA16 | 2,895,226 | 36.55 | 95.32 | 11.63 | 38.10 | 2.67 |
| SA17 | 2,856,203 | 35.91 | 96.16 | 10.03 | 38.22 | 4.35 |
| SA18 | 2,601,183 | 36.31 | 95.46 | 9.83 | 36.73 | 3.61 |
| SA19 | 3,025,090 | 43.13 | 95.64 | 12.03 | 33.76 | 3.38 |
| GT1 | 3,102,325 | 36.05 | 95.98 | 11.97 | 38.48 | 4.95 |
| GT2 | 2,879,369 | 36.69 | 96.09 | 10.80 | 39.35 | 5.04 |
| GT3 | 3,306,596 | 36.16 | 96.09 | 13.60 | 36.87 | 4.83 |
| GT4 | 3,673,088 | 36.01 | 95.87 | 14.44 | 38.68 | 4.56 |
| GT5 | 2,787,415 | 36.29 | 96.01 | 11.32 | 37.33 | 5.07 |
| GT6 | 2,794,396 | 36.42 | 95.91 | 11.00 | 37.47 | 4.86 |
| GT7 | 3,768,890 | 36.20 | 96.27 | 14.74 | 39.31 | 5.06 |
| GT8 | 4,083,564 | 35.79 | 96.12 | 15.52 | 39.67 | 4.20 |
| GT9 | 3,647,831 | 36.22 | 95.78 | 13.78 | 40.76 | 5.15 |
| GT10 | 2,775,456 | 35.99 | 95.82 | 11.30 | 36.83 | 5.02 |
| GT11 | 2,886,971 | 36.32 | 95.65 | 11.92 | 36.79 | 4.94 |
| GT12 | 2,901,809 | 36.39 | 95.11 | 11.59 | 37.94 | 5.25 |
| GT13 | 3,112,640 | 35.91 | 96.00 | 13.65 | 35.10 | 4.75 |
| GT14 | 2,859,771 | 36.14 | 96.15 | 11.29 | 37.19 | 4.95 |
| GT15 | 3,675,384 | 36.33 | 96.21 | 13.77 | 40.04 | 5.13 |
| GT16 | 3,579,736 | 36.44 | 95.88 | 13.42 | 40.57 | 5.50 |
| GT17 | 3,604,498 | 36.47 | 95.75 | 12.87 | 43.55 | 7.00 |
| GT18 | 3,096,731 | 36.16 | 94.95 | 12.34 | 38.33 | 4.81 |
| GT19 | 3,424,117 | 36.31 | 95.58 | 13.24 | 40.62 | 5.36 |
| GT20 | 4,462,078 | 36.08 | 95.90 | 15.92 | 42.55 | 5.18 |
| GT21 | 5,710,033 | 35.99 | 95.57 | 19.30 | 45.45 | 6.45 |
| SZ1 | 3,224,891 | 37.89 | 95.72 | 13.44 | 32.90 | 4.56 |
| SZ2 | 3,417,853 | 38.07 | 95.63 | 18.33 | 29.76 | 3.56 |
| SZ3 | 5,721,738 | 38.05 | 95.50 | 26.59 | 25.85 | 5.07 |
| SZ4 | 3,133,807 | 38.06 | 95.49 | 16.60 | 28.94 | 3.94 |
| SZ5 | 5,115,522 | 37.78 | 96.02 | 26.30 | 31.11 | 2.34 |
| SZ6 | 2,135,159 | 37.79 | 95.74 | 14.31 | 19.81 | 2.53 |
| SZ7 | 3,280,793 | 37.62 | 96.56 | 19.47 | 26.74 | 3.62 |
| SZ8 | 3,902,861 | 37.21 | 95.79 | 18.97 | 32.34 | 4.04 |
| SZ9 | 2,856,891 | 37.44 | 95.15 | 13.67 | 28.09 | 4.13 |
| SZ10 | 3,331,857 | 37.03 | 95.04 | 14.68 | 31.17 | 5.74 |
| SZ11 | 3,337,402 | 36.30 | 95.81 | 14.52 | 35.66 | 4.43 |
| SZ12 | 4,807,683 | 37.60 | 95.59 | 21.23 | 35.43 | 4.45 |
| SZ13 | 4,248,064 | 37.75 | 95.37 | 21.08 | 31.30 | 5.16 |
| SZ14 | 5,883,213 | 38.06 | 95.61 | 24.82 | 28.62 | 6.01 |
| TP1 | 3,414,526 | 35.97 | 96.02 | 11.64 | 40.96 | 6.90 |
| TP2 | 2,987,742 | 36.30 | 96.24 | 11.03 | 40.90 | 6.90 |
| TP3 | 3,053,994 | 36.39 | 96.15 | 11.73 | 39.07 | 5.33 |
| TP4 | 3,202,127 | 35.81 | 95.87 | 11.79 | 40.96 | 7.19 |
| TP5 | 2,508,833 | 35.89 | 96.20 | 9.24 | 40.82 | 7.65 |
| TP6 | 3,039,123 | 36.06 | 95.85 | 11.19 | 40.93 | 8.09 |
| TP7 | 1,683,189 | 35.94 | 95.47 | 7.83 | 33.00 | 4.46 |
| Rice (C) | 1,530,845 | 39.92 | 94.60 | - | - | - |
| Total | - | - | - | 806.1252 | 100 | - |

Table S3 Genetic diversity at seven studied locations based on 52,318 neutral loci and 521 high *F*_ST_ outlier loci, including the percentage of polymorphic loci (Poly%), nucleotide diversity (Pi), observed heterozygosity (*H*_O_), expected heterozygosity (*H*_E_). * indicates significant differences in means between native QL/BM and introduced SY/SA/GT/SZ/TP according to t-tests.

| Dataset | Location | Poly% | Pi | *H*_O_ | *H*_E_ |
| --- | --- | --- | --- | --- | --- |
| Neutral  loci | QL | 89.2 | 0.244 | 0.229 | 0.273 |
|  | BM | 89.4 | 0.285 | 0.368 | 0.318 |
|  | SY | 61.5 | 0.196 | 0.372 | 0.318 |
|  | SA | 45.2 | 0.125 | 0.322 | 0.276 |
|  | GT | 64.0 | 0.205 | 0.296 | 0.320 |
|  | SZ | 59.8 | 0.189 | 0.308 | 0.315 |
|  | TP | 51.1 | 0.194 | 0.382 | 0.380 |
|  | *p* in t-test | 0.003* | 0.026* | 0.459 | 0.425 |
| High *F*_ST_  outlier loci | QL | 46.8 | 0.079 | 0.119 | 0.168 |
|  | BM | 56.0 | 0.147 | 0.299 | 0.262 |
|  | SY | 24.0 | 0.026 | 0.098 | 0.107 |
|  | SA | 30.4 | 0.027 | 0.094 | 0.090 |
|  | GT | 24.7 | 0.032 | 0.113 | 0.130 |
|  | SZ | 24.7 | 0.029 | 0.127 | 0.118 |
|  | TP | 14.0 | 0.043 | 0.244 | 0.303 |
|  | *p* in t-test | 0.003* | 0.007* | 0.318 | 0.391 |

Table S4 Result of the analysis of molecular variation (AMOVA) based on all 53,032 loci, 52,318 neutral loci and 521 high *F*_ST_ outlier loci for the testing of K = 4 (QL+BM+TP, SY+SA, GT, SZ), K = 5 (QL+BM, SY+SA, GT, SZ, TP) and K = 6 (QL+BM, SY, SA, GT, SZ, TP in all 53,032 loci and 52,318 neutral loci) structures. K = 6 based on 521 high *F*_ST_ outlier loci was not tested because of the potential overfitting effect in SA (explained in the text).

| K | Structure | *d.f.* | Sum of squares | Variance | Percentage of v. | *p* |
| --- | --- | --- | --- | --- | --- | --- |
| Total loci | | | | | | |
| 4 | Among groups | 3 | 16,687.17 | 19.51 | 4.29 | 0.2776 |
|  | Among populations  within groups | 3 | 11,911.88 | 108.77 | 23.93 | <0.0001 |
|  | Among individuals  within populations | 121 | 34,599.57 | -40.40 | -8.89 | 1.0000 |
|  | Within individuals | 128 | 46,943.00 | 366.74 | 80.67 | <0.0001 |
| 5 | Among groups | 4 | 18,281.24 | 8.69 | 1.92 | 0.4360 |
|  | Among populations  within groups | 2 | 10,317.81 | 117.29 | 25.93 | <0.0001 |
|  | Among individuals  within populations | 121 | 34,599.57 | -40.40 | -8.93 | 1.0000 |
|  | Within individuals | 128 | 46,943.00 | 366.74 | 81.08 | <0.0001 |
| 6 | Among groups | 5 | 27,909.20 | 123.87 | 26.94 | 0.0489 |
|  | Among populations  within groups | 1 | 689.84 | 9.55 | 2.08 | <0.0001 |
|  | Among individuals  within populations | 121 | 34,599.57 | -40.40 | -8.79 | 1.0000 |
|  | Within individuals | 128 | 46,943.00 | 366.74 | 79.77 | <0.0001 |
| Neutral loci | | | | | | |
| 4 | Among groups | 3 | 16,502.79 | 19.59 | 4.36 | 0.2708 |
|  | Among populations  within groups | 3 | 11,733.04 | 107.12 | 23.86 | <0.0001 |
|  | Among individuals  within populations | 121 | 34,160.91 | -39.88 | -8.88 | 1.0000 |
|  | Within individuals | 128 | 46,347.50 | 362.09 | 80.66 | <0.0001 |
| 5 | Among groups | 4 | 18,092.13 | 9.18 | 2.06 | 0.4096 |
|  | Among populations  within groups | 2 | 10,143.70 | 115.28 | 25.81 | <0.0001 |
|  | Among individuals  within populations | 121 | 34,160.91 | -39.88 | -8.93 | 1.0000 |
|  | Within individuals | 128 | 46,347.50 | 362.09 | 81.06 | <0.0001 |
| 6 | Among groups | 5 | 27,552.16 | 122.23 | 26.93 | 0.0489 |
|  | Among populations  within groups | 1 | 68,3.66 | 9.49 | 2.09 | <0.0001 |
|  | Among individuals  within populations | 121 | 34,160.91 | -39.88 | -8.79 | 1.0000 |
|  | Within individuals | 128 | 46,347.50 | 362.09 | 79.77 | <0.0001 |
| High *F*_ST_ outlier loci | | | | | | |
| 4 | Among groups | 3 | 162.25 | -0.10 | -5.93 | 0.4409 |
|  | Among populations  within groups | 3 | 161.30 | 1.58 | 91.83 | <0.0001 |
|  | Among individuals  within populations | 121 | 25.63 | -0.03 | -1.79 | 0.9853 |
|  | Within individuals | 128 | 35.00 | 0.27 | 15.88 | <0.0001 |
| 5 | Among groups | 4 | 163.35 | -0.49 | -29.21 | 0.6051 |
|  | Among populations  within groups | 2 | 160.19 | 1.92 | 114.74 | <0.0001 |
|  | Among individuals  within populations | 121 | 25.63 | -0.03 | -1.84 | 0.9971 |
|  | Within individuals | 128 | 35.00 | 0.27 | 16.32 | <0.0001 |

Table S5 Pairwise *F*_ST_ values based on 52,318 neutral loci and 521 high *F*_ST_ outlier loci. All pairs values were significantly different for zero with *p* < 0.001, except for the QL-BM pair among the high *F*_ST_ outlier loci (pairwise *F*_ST_ = 0.194, value *p* = 0.721).

| Dataset |  | QL | BM | SY | SA | GT | SZ | TP |
| --- | --- | --- | --- | --- | --- | --- | --- | --- |
| Neutral  loci | QL |  |  |  |  |  |  |  |
|  | BM | 0.017 |  |  |  |  |  |  |
|  | SY | 0.212 | 0.188 |  |  |  |  |  |
|  | SA | 0.317 | 0.313 | 0.465 |  |  |  |  |
|  | GT | 0.183 | 0.174 | 0.348 | 0.452 |  |  |  |
|  | SZ | 0.225 | 0.195 | 0.209 | 0.496 | 0.363 |  |  |
|  | TP | 0.127 | 0.115 | 0.309 | 0.456 | 0.291 | 0.329 |  |
| High *F*_ST_  outlier loci | QL |  |  |  |  |  |  |  |
|  | BM | 0.194 |  |  |  |  |  |  |
|  | SY | 0.890 | 0.756 |  |  |  |  |  |
|  | SA | 0.938 | 0.884 | 0.960 |  |  |  |  |
|  | GT | 0.222 | 0.283 | 0.953 | 0.966 |  |  |  |
|  | SZ | 0.804 | 0.613 | 0.096 | 0.930 | 0.876 |  |  |
|  | TP | 0.186 | 0.094 | 0.882 | 0.937 | 0.520 | 0.709 |  |
